# Rhabdomyolysis due to Trimethoprim-Sulfamethoxazole Administration following a Hematopoietic Stem Cell Transplant

**DOI:** 10.1155/2015/619473

**Published:** 2015-10-18

**Authors:** Alexander Augustyn, Mona Lisa Alattar, Harris Naina

**Affiliations:** ^1^Hamon Center for Therapeutic Oncology Research, University of Texas Southwestern Medical Center, Dallas, TX 75390, USA; ^2^Simmons Comprehensive Cancer Center, University of Texas Southwestern Medical Center, Dallas, TX 75390, USA; ^3^Department of Hematology and Oncology, University of Texas Southwestern Medical Center, Dallas, TX 75390, USA

## Abstract

Rhabdomyolysis, a syndrome of muscle necrosis, is a life-threatening event. Here we describe the case of a patient with chronic myeloid leukemia who underwent a haploidentical stem cell transplant and subsequently developed rhabdomyolysis after beginning trimethoprim-sulfamethoxazole (TMP/SMX) prophylaxis therapy. Rechallenge with TMP/SMX resulted in a repeat episode of rhabdomyolysis and confirmed the association. Withdrawal of TMP/SMX led to sustained normalization of creatine kinase levels in the patient. A high index of suspicion is necessary to identify TMP/SMX as the cause of rhabdomyolysis in immunocompromised patients.

## 1. Introduction

Rhabdomyolysis is a potentially life-threatening syndrome of muscle necrosis characterized by the release of intracellular muscle contents into the systemic circulation and can result in significant muscle pain, electrolyte imbalance, acute renal failure, and even death [1, 2]. Many medications, including salicylates, statins, neuroleptics, and fibrates, are associated with rhabdomyolysis although few reports indicate trimethoprim-sulfamethoxazole (TMP/SMX), a commonly used antibiotic, as the culprit [2–8]. Here we describe the case of a patient with blast phase chronic myeloid leukemia and subsequent haploidentical stem cell transplant maintained on dasatinib who developed rhabdomyolysis when concurrent TMP/SMX prophylaxis was initiated.

The classic triad of rhabdomyolysis includes muscle pain, weakness, and dark urine although the presentation can vary from asymptomatic elevations of muscle enzymes to severe muscle pain with acute kidney failure [1, 2]. In addition to characteristic symptoms, about half of patients also present with myoglobinuria, while more severe cases can present with electrolyte imbalances such as hyperkalemia, acute renal failure, and/or swelling of the extremities [9, 10]. The trademark laboratory diagnosis is an elevation of creatine phosphokinase (CK) to levels 5 times the normal limit, with a range of approximately 1,000 to 100,000 international units per liter (IU) [11].

The association of TMP/SMX with rhabdomyolysis is rare, and most cases have been reported in patients with human immunodeficiency virus (HIV) who receive TMP/SMX as prophylaxis against* Toxoplasma gondii* and prophylaxis or treatment for* Pneumocystis jirovecii* pneumonia (PJP) [3, 4, 6, 7]. TMP/SMX was also reported as the cause of rhabdomyolysis in one patient with CML who subsequently underwent an unrelated donor allogeneic stem cell transplant, developed PJP, and was treated with high-dose TMP/SMX although without concurrent tyrosine kinase inhibitor (TKI) therapy [5]. A diagnosis of rhabdomyolysis was made after the patient developed lactic acidosis, acute renal failure, and hypotension with dramatic elevation of CK levels. Discontinuation of TMP/SMX led to CK normalization within five days [5]. Here, we report the case of a patient with CML and haploidentical stem cell transplant who developed rhabdomyolysis while receiving TMP/SMX for PJP prophylaxis. Discontinuation of all medications resulted in CK normalization while the rechallenge with TMP/SMX caused repeated elevation of CK levels, supporting the diagnosis.

## 2. Case Presentation

A 28-year-old male with a past medical history significant only for benign hypertension presented at our institution for swelling of the left mandible in 2011. Routine blood work revealed a white blood cell count (WBC) of 298,000 with 2% blasts. Peripheral blood polymerase chain reaction (PCR) was positive for the t(9; 22) BCR-ABL translocation. The patient was started on imatinib after bone marrow biopsy confirmed the diagnosis of chronic myeloid leukemia, chronic phase (CML-CP). He initially achieved a complete hematologic response but six months later was found to have a WBC of 59,000 with 37% blasts and an elevated lactate dehydrogenase. Bone marrow biopsy revealed a mixed phenotype acute leukemia (B-cell/myeloid) most consistent with CML in blast phase. Due to progression on imatinib, he was treated with the R-hyper-CVAD regimen plus dasatinib while awaiting bone marrow transplantation.

Two years later, in January 2013, our patient received a haploidentical transplant and his course was free from graft versus host disease and major infections. He achieved major molecular response and was maintained on dasatinib. Six months after transplantation, his cytopenias resolved, immunosuppressive agents were tapered completely, and he was started on TMP/SMX and valacyclovir prophylaxis. Of note, the patient did not use any herbal remedies.

In September of 2013, the dasatinib dose was increased from 75 mg daily to 100 mg after tacrolimus was discontinued and he received five vaccinations (influenza, TDaP, HepB, Hib, and IPV). Four days later, our patient presented at his usual follow-up clinic visit with complaints of dark urine despite adequate water intake with no diarrhea or other symptoms. He did not report any abnormal exercise routines. Initial laboratory evaluation revealed LDH 3172 international units/L (IU/L), AST 1532 IU/L, and ALT 321 IU/L. The patient's baseline AST and ALT were 22 IU/L and 21 IU/L, respectively, measured three months prior to admission. Immediately, all medications including dasatinib, TMP/SMX, amlodipine, valacyclovir, and pantoprazole were discontinued. CK was found to be markedly elevated at 132,400 IU/L. Fluids were administered and his CK dropped to 76,600 IU/L overnight; he was discharged one day later with CK at 43,700 IU/L along with instructions to avoid strenuous exercise and be followed up closely in the clinic. 11 days later, his CK levels normalized at 502 IU/L and the decision was made to restart dasatinib at 100 mg per day. No other medications were restarted. Four days later, his CK was measured at 301 IU/L, and PJP prophylaxis with TMP/SMX was restarted. One week later, the patient presented for a scheduled laboratory workup and was found to have a CK of 34,300 IU/L but was otherwise asymptomatic, with clear yellow urine.

**Figure 1:**
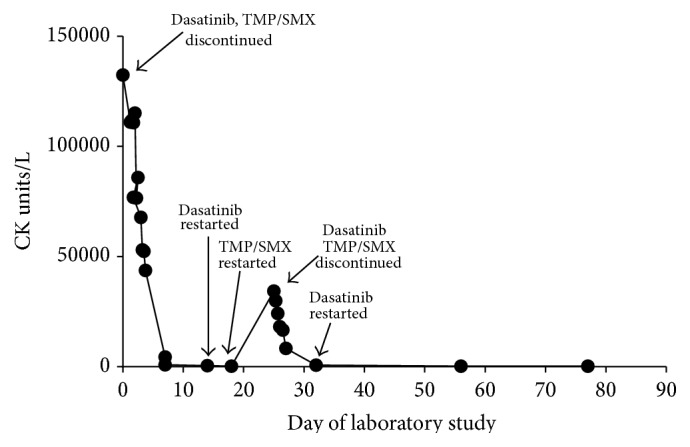
Plot of creatine kinase levels versus day of laboratory study. Relationship of creatine kinase levels to medication administration or cessation is indicated by arrows.

The patient was admitted and TMP/SMX and dasatinib were once again held, fluids were administered, and his CK levels decreased to 8,300 IU/L when he was discharged two days later. Due to the temporal association of CK elevation following rechallenge with TMP/SMX, the decision was made to not provide prophylaxis for PJP. The patient was continued on valacyclovir and dasatinib. Since TMP/SMX was completely stopped, his CK levels have remained normal (Figure 1). Complete medication dosing and CK, LDH, AST, and ALT levels for both inpatient hospitalizations are provided in Table 1.

## 3. Discussion

In a study involving 475 patients with rhabdomyolysis, exogenous toxins including medically administered drugs, alcohol, and illicit substances were determined to be the cause in 46% of cases [11]. Prior cases of TMP/SMX-induced rhabdomyolysis have occurred in patients with HIV receiving prophylaxis or treatment for toxoplasmosis or PJP [3, 4, 6, 7]. One prior report detailed rhabdomyolysis in a patient with AML who underwent allogeneic stem cell transplant and developed PJP, necessitating treatment with TMP/SMX [5]. In our patient, before the discovery of TMP/SMX as the likely causative agent of rhabdomyolysis, we considered other etiologies, such as dasatinib, vaccination, or extreme exercise. Dasatinib use has been associated with rare occurrences of rhabdomyolysis (<1% of patients), according to the official drug data sheet, although no case reports currently detail such an association [12]. Vaccines for influenza and TDaP have also been temporally associated with the development of rhabdomyolysis in isolated case reports [13–15]. Based on the Naranjo probability scale of adverse drug reactions, TMP/SMX was the likely causative agent of rhabdomyolysis in our patient with a score of 6 (probable adverse drug reaction) [16]. This was confirmed by rechallenge with TMP/SMX, which resulted in elevation of CK to over 30,000 IU/L.

Several reports implicated imatinib as the cause of rhabdomyolysis. These patients were treated with imatinib for CML and aggressive fibromatosis [17–19]. In each case, withdrawal of imatinib or transition from imatinib to the second-generation tyrosine kinase inhibitor dasatinib resulted in resolution of rhabdomyolysis. Gordon et al. also identified a high number of CK abnormalities in patients treated with imatinib for CML or gastrointestinal stromal tumors, suggesting that this drug is associated with rare development of severe rhabdomyolysis [19]. However, to date, no report has directly linked dasatinib to rhabdomyolysis, and this remains true in the case of our patient whose CK levels have remained within normal limits on dasatinib maintenance therapy.

Drug-drug interactions such as those identified between cytochrome P450 isoform 3A4 inhibitors and HMG-CoA reductase inhibitors (statins) are known to cause rhabdomyolysis. For example, cotreatment with simvastatin and fluconazole, a known CYP isoenzyme 3A4 (CYP3A4) inhibitor, can cause rhabdomyolysis in patients likely due to elevated plasma levels of simvastatin [20]. Dasatinib is metabolized primarily by CYP3A4 and is a known time-dependent inhibitor of CYP3A4 [12, 21, 22]. TMP/SMX is a potent inhibitor of CYP2C8 and CYP2C9 and also inhibits CYP3A4 at higher concentrations [23]. However, the steady state plasma concentrations of both TMP (approximately 6 *μ*M) and SMX (approximately 270 *μ*M) are below that required to appreciably inhibit CYP3A4 in human cells (over 250 *μ*M for TMP, over 500 *μ*M for SMX, resp.), suggesting that a drug-drug interaction elevating levels of TMP/SMX and/or dasatinib leading to rhabdomyolysis is unlikely [23, 24]. Of course, wide variability exists in cytochrome P450 enzymatic capacity among humans, so this possibility cannot be completely excluded at the present time [25]. The occurrence of drug-drug interactions increases as the number of medications increases and factors such as gastrointestinal absorption, drug distribution, and drug metabolism can enhance this effect [26]. Further study is needed to determine if a drug-drug interaction occurs between dasatinib and TMP/SMX, especially since both drugs are known to modulate CYP family members* in vitro*. If such an interaction is found to occur, pentamidine may be the preferred mode of PJP prophylaxis instead of TMP/SMX in the setting of concurrent TKI usage.

## Figures and Tables

**Table 1:** Complete medication and dosing history with relevant laboratory values for our patient during first inpatient admission for rhabdomyolysis (September 9, 2013), discharge (September 12, 2013), and outpatient clinic follow-up visit (September 27, 2013). The same information is also presented for the second inpatient admission for rhabdomyolysis (October 3, 2013), discharge (October 6, 2013), and outpatient clinic follow-up visit (October 14, 2013).

	September 9	September 12	September 27	October 3	October 6	October 14
Medications						
Dasatinib	100 mg daily	—	100 mg daily	100 mg daily	100 mg daily	100 mg daily
TMP/SMX	160/800 MWF	—	160/800 MWF	160/800 MWF	—	—
Valacyclovir	500 mg daily	—	—	—	—	500 mg daily
Amlodipine	5 mg daily	—	5 mg daily	5 mg daily	5 mg daily	5 mg daily
Pantoprazole	40 mg daily	—	40 mg daily	40 mg daily	40 mg daily	40 mg daily
Lab values (IU/L)						
CK	132,400	43,700	301	34,308	8,329	285
LDH	3,172	—	203	647	—	210
AST	1,532	978	26	535	198	26
ALT	321	351	28	230	149	37
